# Effects of short-term environmental stresses on the onset of cannabinoid production in young immature flowers of industrial hemp (*Cannabis sativa* L.)

**DOI:** 10.1186/s42238-021-00111-y

**Published:** 2022-01-04

**Authors:** Sang-Hyuck Park, Christopher S. Pauli, Eric L. Gostin, S. Kyle Staples, Dustin Seifried, Chad Kinney, Brian D. Vanden Heuvel

**Affiliations:** 1 Institute of Cannabis Research, Colorado State University–Pueblo, 2200 Bonforte Blvd., Pueblo, CO 81001 USA; 2 Department of Biology, Colorado State University–Pueblo, 2200 Bonforte Blvd., Pueblo, CO 81001 USA; 3 Department of Chemistry, Colorado State University–Pueblo, 2200 Bonforte Blvd., Pueblo, CO 81001 USA

**Keywords:** *Cannabis sativa*, Cannabinoids, CBD, THC, Abiotic stress, Biotic stress, Secondary metabolites

## Abstract

**Backgrounds:**

*Cannabis sativa* L. produces at least 120 cannabinoids. Although genetic variation is the main factor in cannabinoid production, the effects of short-term environmental stresses in the early flowering stage remains largely unknown.

**Methods:**

To investigate the effects of short-term environmental stresses on the onset of cannabinoid production in young immature flowers, a hemp variety, Green-Thunder (5–8% CBD/mg of dry weight), was treated with mechanical damage, insect herbivory, extreme heat, or drought stress for 5–7 days during the first 2 weeks of flowering. Three hemp tissues, including flowers, leaves, and stems, were collected from hemp grown under these stress conditions at multiple time points during the first 2 weeks after transition to the short photoperiod and analyzed using high pressure liquid chromatography to quantify phytocannabinoids including cannabigerolic acid (CBGA), cannabigerol (CBG), cannabidiolic acid (CBDA), cannabidiol (CBD), Δ-tetrahydrocannabinolic acid (THCA), Δ-tetrahydrocannabinol (THC), and cannabinol (CBN).

**Results:**

The 5 days of mechanical wounding did not affect the production of any of the cannabinoids during the initial stage of flowering. However, after 5 days of herbivore treatment, there was a significant difference in concentration between day 1 and day 6 of CBGA (control: 308 μg/g; treatment – 24 μg/g), CBG (control: 69 μg/g; treatment: 52 μg/g), and CBD (control: 755 μg/g; treatment: 194 μg/g) between the control and treatment plants. The 7 days of heat treatment at 45–50 ^o^C significantly reduced the production of CBGA during this observed window (control: 206 μg/g; treatment: 182 μg/g) and CBG (control: 21 μg/g; treatment: − 112 μg/g). Notably, the largest change was observed after 7 days of drought stress, when plants showed a 40% greater accumulation of CBG (control: 336 μg/g; treatment: 622 μg/g), and a significant decrease (70–80%) in CBD (control: 1182 μg/g; treatment: 297 μg/g) and THC amounts (control: 3927 μg/g; treatment: 580 μg/g).

**Conclusions:**

Although this observation is limited in the early flowering stage, the common field stresses are adequate to induce changes in the cannabinoid profiles, particularly drought stress being the most impactful stress for hemp flower initiation with the altering the cannabinoid production by decreasing CBD and THC accumulation while increasing CBG by 40%.

**Supplementary Information:**

The online version contains supplementary material available at 10.1186/s42238-021-00111-y.

## Introduction

Plants produce secondary metabolites that play important roles in plant adaptation and survival under stress conditions along with primary metabolites that are essential for their growth and development (Ramakrishna and Ravishankar 2011). Often, the secondary metabolites confer specific odors, colors, and tastes in plants. Plant secondary metabolites have been widely used as sources for food additives, flavors, cosmetics, industrial biochemicals, and biopharmaceuticals (Gonçalves et al. 2019; Ramakrishna and Ravishankar 2011).

*Cannabis sativa* L., a member of the Cannabaceae family, is a primarily annual, dioecious variety that produces secondary metabolites called cannabinoids (Elsohly and Slade 2005; White et al. 2016). *Cannabis* produces at least 120 different cannabinoids which are synthesized in the basal disk cells and stored in the secretory cavity in glandular trichomes (Ebersbach et al. 2018; Livingston et al. 2020). The different cannabinoid carrying capacity observed in *Cannabis* is largely dependent on the number of trichomes (Kim and Mahlberg 1997; Livingston et al. 2020). Flower tissues have the highest concentration of glandular trichomes, which corresponds to this tissue showing the greatest amounts of cannabinoids. Two major cannabinoids are (-)-Δ^9^-trans-tetrahydrocannabinolic acid (Δ^9^–THCA) and cannabidiolic acid (CBDA) that are synthesized from a common precursor, cannabigerolic acid (CBGA), by THCA and CBDA synthase respectively, and then can be decarboxylated into CBD and THC through various processes, such as heating, light exposure, or through chemical reactions (Chandra et al. 2017; Wang et al. 2016).

Over the past years, *Cannabis* plants, especially its chemical derivatives cannabinoids, have regained public attention due to their therapeutic potentials in Dravet and Lennox-Gastaut syndrome, Parkinson’s disease, cancers, mental illnesses, and other neurological disorders (Montoya et al. 2020; Pauli et al. 2020). In the 2018 Farm Bill, hemp has been reclassified as an agriculture commodity. To meet increased demands, a great deal of selective breeding efforts has been made to develop elite varieties with various cannabinoid profiles and new dominant cannabinoids (de Meijer and Hammond 2005; de Meijer et al. 2009a, b; Williamson 2004). Furthermore, yeast has successfully been genetically engineered as an alternative cannabinoid production platform (Luo et al. 2019).

The phytocannabinoid content of *Cannabis* is primarily determined by genetic factors that are inheritable and differentially expressed dependent on variety, tissue type, position of tissue, and the growth stage (Chandra et al. 2013; Kovalchuk et al. 2020; Namdar et al. 2018). In addition, environmental conditions are the other major factors that contribute to the production and accumulation of cannabinoids (Gorelick and Bernstein 2017). Like other crops, the production of cannabinoids, especially THC and CBD, are also greatly affected by a variety of environmental stresses such as light (Eichhorn Bilodeau et al. 2019; Magagnini et al. 2018), temperature (Gorelick and Bernstein 2017), water deficit (Caplan et al. 2019; Gorelick and Bernstein 2017), nutrition (Bernstein et al. 2019a, Gorelick and Bernstein 2017), heavy metals (Husain et al. 2019), phytohormones (Burgel et al. 2020), soil bacteria (Pagnani et al. 2018), and biotic stresses including insect and microbial pathogens (Langenheim 1994; McPartland et al. 2000; Sirikantaramas et al. 2005). Environmental stresses cause numerous plant-wide responses, which are classified into three types: (1) systematic acquired resistance to pathogens (Fu and Dong 2013), (2) systematic wound responses to damage and herbivory (Savatin et al. 2014), and (3) systemic acquired acclimation to abiotic stresses (Mittler and Blumwald 2015). A recent study shows that traits like the number of days to maturity as well as THC and CBD production are strongly determined entirely by genetic factors, whereas traits such as plant height, grain yield, and water usage were influenced by environmental factors and the interaction between genetic and environmental factors (Campbell et al. 2019).

Abiotic factors or elicitors include photon-radiation, nutrient deficiency, heavy metal presence, drought, and temperature stress (Gorelick and Bernstein 2017). *Cannabis* plants have evolved in high irradiation environments which plays a critical role in leaf morphology and the production of cannabinoids (Magagnini et al. 2018). Magagnini et al. 2018 showed that *Cannabis* grown under blue wavelength (~ 450–520 nm) with a short photoperiod (12 h light/12 h dark) yielded the highest CBD and THC accumulations. Also, the blue light spectrum induced a greater amount of cannabigerol (CBG) when combined with UV-A light (Eichhorn Bilodeau et al. 2019; Magagnini et al. 2018). This was also supported by a more recent study, where the authors observed the greatest cannabinoid production change by blue-rich light spectrums, specifically observing CBGA accumulation stimulation when comparing to far-red lights (Danziger and Bernstein 2021a). In long-day plants, flowering is mostly promoted by red (R, ~ 625–700 nm) and far-red (FR, > 700 nm) lights which is delivered during the early and later parts of the photoperiod, respectively (Backer et al. 2019; Eichhorn Bilodeau et al. 2019). However, the response of CBDA, THCA, and CBCA to light spectrum appeared to be cultivar-specific (Danziger and Bernstein 2021a). Some *Cannabis* genotypes such as “G-170” appeared to be insensitive to changes in the R:FR, showing no effect on flowering in response to the changes in R:FR ratio (Magagnini et al. 2018). The same study additionally showed that the light insensitive *Cannabis* has an increased development of mature cuttings when exposed to the low R:FR ratio during a long photoperiod (18 h light/6 h dark) (Magagnini et al. 2018).

In addition, temperature is an important factor for canopy formation in fiber hemp. A study showed that the rate of leaf appearance and stem elongation linearly increased as temperature rises from 10 ^o^C to 28 ^o^C (Van Der Werf et al. 1995). Due to global warming, agricultural crops including *Cannabis* plants encounter multiplexed environmental stresses of which extreme heat and drought are the most impactful. Herppich et al. 2020 showed that two fiber-type hemp varieties (‘Santhica 27’ and ‘Ivory’) responded differently against the combined stress. ‘Santhica 27’ utilized low CO_2_ uptake rates in lower leaves and higher seed germination rates that resulted in twice the fiber yield compared to ‘Ivory’, whereas ‘Ivory’ developed high rates of CO_2_ gain which enabled the establishment of large leaf area with more stomatal regulation, resulting in higher water use efficiency (Herppich et al. 2020). As seen in this study, fiber hemp promptly reprogramed their photosynthetic rate, transpiration, stomatal regulation, and water use efficiency upon treatment of environmental stresses, affecting the yield performance, which affects the total content and potentially the ratios of secondary metabolites in *Cannabis*.

Another recent study demonstrated that drought stress significantly alters gene expression patterns (Gao et al. 2018). Through analyzing differential gene expression of drought stress compared to a control group, it appears there were 55 differentially expressed genes that are responsible for secondary metabolite biosynthesis in response to drought conditions, as well as the genes involved in the ABA induction and its signal transduction pathway; however, chemical concentrations were not measured to identify if the observed gene expressions differences affected secondary metabolite production (Gao et al. 2018).

Mineral nutrient supplements can impact cannabinoid profiles as well. It has been reported that the supplementation of mineral nutrients (e.g., nitrogen, calcium, iron, and magnesium) increased cannabinoids production, although others have reported that nutrient deficiencies may stimulate the cannabinoid production as well (Gorelick and Bernstein 2017). A study showed that phosphorus enhanced fertilizer increases the level of CBD, CBG, CBN while decreasing THC by 16% (Bernstein et al. 2019a). A more recent study in 2021 demonstrated the dose-dependent effects of phosphorus in cannabinoid biosynthesis as well as plant functional-physiology, which affects the pharmacological quality of cannabinoids (Shipony and Bernstein 2021). Another key nutrient, nitrogen, also significantly affected the *Cannabis* chemical profile. A recent study showed that the highest concentrations of cannabinoid and terpenoids were highest under the 30 mg/L of nitrogen and reduced with the increase of nitrogen supply (Saloner and Bernstein 2020). Three inorganic nutrients, nitrogen, phosphorus, and potassium, supplemented with humic acids significantly increased the level of CBG by 71% in flowers while lowering CBN levels by 38% and 36% in both flowers and inflorescence leaves, respectively (Bernstein et al. 2019a). Heavy metals can also affect plant physiology and molecular mechanisms. *Cannabis* plants are tolerant to moderate concentration of cadmium (Linger et al. 2005), nickel, and chromium (Citterio et al. 2005), with no major effects on growth and development (Gorelick and Bernstein 2017). A recent study indicated that six industrial hemp varieties grown on the abandoned coal mine soil in Pennsylvania had a significant increase level of total CBD content when compared to those grown in a commercial soil while the seed germination rates and plant height were not affected (Husain et al. 2019).

Additionally, phytohormones are important growth regulators which play an important role in the plant cellular processes, metabolism, growth, and plant defense responses against stresses (Egamberdieva et al. 2017, Gorelick and Bernstein 2017). Physical stressors (e.g., rainstorms, hailstorms, herbivory, and mechanical damages from agricultural equipment) generally found in hemp fields induce signal molecules such as jasmonic acid (JA), ABA, systemin, and oligogalacturonides, alerting both adjacent and remote tissues to activate mechanisms required for healing and further defense (Leon et al. 2001). Upon receiving a signal molecule, various enzymatic pathways are modulated to alter contents of bioactive metabolites such as terpenoids, alkaloids, and phenylpropanoids (Chandra et al. 2017).

To elucidate the roles of endogenous hormones, a *Cannabis* suspension cell culture was used to study the application of JA and salicylic acid (SA). The results showed no significant changes in cannabinoid content, which might be due to the limited amount of cannabinoid production in the cell culture system (Flores-Sanchez et al. 2009; Peč et al. 2010). However, a study performed on drug-type *Cannabis* plants with a abscisic acid (ABA) treatment showed the reduction of CBD and THC levels during vegetative stage (Mansouri and Asrar 2012) while increased THC was observed in leaves and flowers in male plants (Mansouri et al. 2009; Mansouri and Asrar 2012).

Several studies also reported that exogenously applied plant growth regulators have an impact on morphology, flower biomass, and cannabinoid contents of *Cannabis sativa* L. (Burgel et al. 2020; Lalge et al. 2016). A study showed that the plant architecture (e.g., height, length of axillary branches, and number of internodes) was significantly reduced by the treatment of synthetic auxin (1-naphthalenaecetic acid; NAA), cytokine (6-benzylaminopurine; BAP), and a mixture of both, when compared to the untreated control plants (Burgel et al. 2020). It is noted that BAP reduced height and number of internodes but the length of axillary branches were not affected. These exogenously applied growth regulators on the hemp variety ‘KANADA’ have aided in the uniform and compact growth of the plants, as well as enabled higher CBD production in indoor growth conditions (Burgel et al. 2020).

Other than aforementioned environmental factors, hemp biomass yield and cannabinoid production can be also influenced by various agronomical practices such as plant density, sowing time, cropping system (García-Tejero et al. 2019), plant architecture (Danziger and Bernstein 2021b), and geographical location (Aubin et al. 2016).

Similar to abiotic stresses, biotic stresses also cause metabolite concentration changes. Hemp plants are susceptible to a variety of insects. Nearly 300 insect pests have been reported but only a few are known to cause economic loss (McPartland 1996). In hemp plants, the most serious pests are lepidopterous stem borers including European corn borers (*Ostrinia nubilalis*) and hemp borers (*Grapholita delineana*) (McPartland 2002). Comparing to other field crops, hemp is relatively pest-tolerant plants because of the pest-repellent properties of cannabinoids and terpenoids present (Dambolena et al. 2016). Recent studies have demonstrated that cannabinoid-enriched extracts show pesticidal activity to defend themselves from insect herbivore attacks (Benelli et al. 2018; Park et al. 2019a). Although the impacts of insect damage on cannabinoid content is not well documented, the increase in biomass and seed productivity were observed in hemp resulting from branch proliferation caused by European corn borer (Small et al. 2007). It is also reported that phytocannabinoid concentration and composition was significantly correlated to structure of endorhiza communities (Winston et al. 2014).

The aim of this study is to investigate if commonly reported biotic and abiotic stresses, including mechanical damage, insect damage, excess heat, and drought stress, influence cannabinoid production and bioaccumulation of a local Colorado hemp variety (*Cannabis sativa* L.) during the early flowering stage under semi-controlled environment. Better understanding on the environmental effects of cannabinoid production would be a necessary step for a successful hemp cultivation.

## Materials and methods

### *Cannabis* cultivation and cloning

For cannabinoid analysis, the local Colorado hemp variety that is known to produce around 5–10% CBD, named Green-Thunder, was grown in a greenhouse maintained at 20–25 ^o^C under a 16 h light/8 h dark cycle at a humidity level of 50–70%. A single female hemp plant was used to generate multiple clones that provided similar cannabinoids levels. To produce clones, an apical meristem with at least one set of fan leaves were taken from the mother plant and treated with Clonex rooting gel (Growth Technology Ltd., Somerset, UK) containing indole-3-butyric acid, then placed into a rapid rooter, which is a moist compressed coconut/peet cube (3 cm length × 3cm width × 5cm height). The cubes were maintained in a hydroponic system (38 cm length × 30 cm width × 26 cm height) that is filled with the root induction solution containing 12 L deionized water blended with 31 ml of Botanicare Liquid Karma (Botanicare, Vancouver, WA) under T5 fluorescent light bulbs (432 W, 242 μmol/m^2^/s) until the roots reached 15–20 cm long. The rooted clones were transplanted into 25 cm square pots filled with Pro-Mix HP soil (PRO-MIX, Quakertown, PA) and acclimated for a week. Then, the clones were moved to a grow tent (2 m length × 1.5 m width × 2 m height) maintained at 20–25 ^o^C at a humidity level of 50–70% under 16 h light/8 h dark cycle during vegetative stage for 6 weeks and then into a 12 h light/12 h dark cycle to induce the flowering stage and simulate each different stress condition. The hemp plants were irrigated with 1 L of water at twice weekly, except for drought treatment (200 ml).

### Stress treatments

For our stress experiment, a total of 12 hemp clones were taken from a 2-month-old mother plant forced to remain at a vegetative stage with 24 h light cycle under AgroBrite T5 fluorescent light bulbs (432 W, 242 μmol/m^2^/s) (Hydrofarm, Denver, CO). The height of the mother plant was controlled by cutting apical and lateral meristems at around 1 m tall. Six untreated control plants were grown under the semi-controlled grow tent (2 m length × 1.5 m width × 2 m height) maintained at 20–25 ^o^C at a humidity level of 50–70% under 16 h light/8 h dark cycle during vegetative stage for 6 weeks, while the other six plants were grown in a separate grow tent under same conditions except for the given stress. For all the stress tests, 7-week-old hemp clones that are in week 2 of the flowering stage were used. For the mechanical wounding experiment, on day 1, three fully expanded fan leaves, at least three nodes from the top were punctured using a 1/4″ round hole punch for 12 holes per leaf. For the main stem wounding, a blade was used to generate five 1-inch-long vertical incisions on the stalk. The percentage of tissue loss per leaf and stems was maintained at no more than 20%. In a series of wounding introduced in the next 2 days, the remaining lower parts of the plant were treated. For herbivore damage, a total of twenty 3rd instar caterpillar larvae of tobacco hornworm *Manduca sexta* were placed on the leaves of six hemp plants that were covered with a mesh net bag for 5 days. For the heat treatment, excess heat was generated by a heater that maintained the grow tent at 45–50 ^o^C for 7 days. To prevent the tent from overheating, an additional exhaust fan was installed for better ventilation. For the drought experiment, the water deficient condition was simulated by irrigating twice a week with 200 ml which is approximately 20% relative water content in the soil, while control plants were grown under well-watered condition. Once each treatment was completed, the flower, leaf, and stem tissues were collected separately. Of six hemp plants initially subjected to each stress test, the three most healthy- and representative plants were chosen for cannabinoid analysis. All flowers, leaves, and stems tissues were separately collected, and the sampled tissues were immediately stored on ice and then stored at -80 ^o^ C until further analysis.

### Analysis of Cannabinoids using high pressure liquid chromatography (HPLC)

The cannabinoid extraction from plant materials was conducted using the United Nations Office on Drugs and Crime (UNODC) method with modifications (UNODC 2009). Samples were weighed to 0.5 g, then extracted with 5 ml of a (9:1, v/v) methanol (MeOH): chloroform solution. The mixture was vortexed before being placed in a sonication bath for 15 min and vortexed every 5 min. The sample was then centrifuged at 4500 rpm for 15 min and then the supernatant was collected and stored in a refrigerator at 4 °C until analysis. Extracts were analyzed by high pressure liquid chromatography (HPLC) on a Dionex UltiMate 3000. Stock standards of cannabigerolic acid (CBGA), cannabigerol (CBG), cannabidiolic acid (CBDA), cannabidiol (CBD), Δ-tetrahydrocannabinolic acid (THCA), Δ-tetrahydrocannabinol (THC), and cannabinol (CBN) were purchased from Cerilliant (Millipore Sigma, St. Louis, MO) in 1mg/ml glass ampules. The stock standard of 100 μg/ml of the cannabinoids was diluted in MeOH which was used to create the calibration curve, where 10 μg/ml, 50 μg/ml, and 100 μg/ml were points on the curve. HPLC analysis included external calibration with each set of samples, solvent blanks, and analysis of independently prepared continuing calibration verification samples every twentieth sample to ensure accuracy. A binary gradient at a flow rate of 0.450 ml/min was used for separation. Mobile phase A was a 0.1% formic acid aqueous solution and mobile phase B consisted of MeOH. Cannabinoids were quantified by absorbance at 210 nm. Chromatographic separation of the cannabinoids was accomplished using an Accucore aQ C18 Polar Endcapped column (100 mm × 2.1 mm, particle size 2.6 μm). Initial conditions: 67% B with a non-linear gradient to 81% B from 0.0 to 8.0 min giving the gradient a concave inflection point near 5.0 min. The remaining gradients were linear. Mobile phase B was increased to 83% at 13.0 min, then increased again to 95% at 16.0 min, and then returned to 67% for 7.0 min of equilibration.

### Statistical analysis

The differences in cannabinoid levels in each tissue (*n* = 3) and the tissue-specific differences over day 7, 12, and 14 during the first 2 weeks of flowering stage were analyzed for statistical significance by one-way ANOVA and two-way ANOVA, respectively, followed by Tukey’s post-test available in the GraphPad Prism software package (GraphPad Software, Inc., La Jolla, CA). Differences between the untreated control and stress-treated groups were considered to be statistically significant at *p < 0.05, **p < 0.01, ***p < 0.001, ****p < 0.0001. All graphs were represented as mean ± standard deviation (s.d.). Concentration values reported are the difference between the concentration on day 1 and the concentration on day 6 or day 8 for each the control and treated plants. A positive value indicates that an increase in that cannabinoid concentration during that timeframe was observed, whereas, a negative value indicates that cannabinoid decreased concentration between days 1 and day 6 or day 8 depending on the treatment.

## Results and discussion

### Early floral cannabinoid production

To determine the spatial- and temporal-specific cannabinoid production, the amounts of CBGA, CBG, CBDA, CBD, THCA, THC, and CBN in the immature buds, leaves, and stem tissues were quantified and compared. A previous study reported that the concentration of most cannabinoids in female flowers are two-fold higher than the concentration in the inflorescence leaves (Bernstein et al. 2019b). In this study, flower tissue showed the highest cannabinoid production among three tissues. In flowers, the production of CBDA and THCA appeared to be the most predominant, producing 11,848 μg/g (1.1% w/w) and 12,028 μg/g (1.2% w/w), respectively (Supplementary Fig. 1). The floral production of CBDA were 2.4-fold higher than the production in leaf tissues (*n* = 24, *p* < 0.0001) and 25-fold higher than the production in stem tissues (*n* = 24, *p* < 0.0001) (Supplementary Fig. 1). Similarly, the production of THCA was 2.3-fold higher production than the production in leaf tissues (*n* = 24, *p* < 0.0001) and 29.8% higher than the production in stem tissues (*n* = 24, *p* < 0.0001) (Supplementary Fig. 1). Other cannabinoids including CBGA, CBG, CBD, and THC were produced less than 0.05%. However, the levels were consistently high in flower tissues than leaf and stem tissues (Supplementary Fig. 1).

Floral cannabinoid production on day 7, 12, and 14 after the transition to the short-day were measured using HPLC. The cannabinoid analyses showed that the amount of CBDA increased 1.5-fold from 11,848 μg/g (1.1% w/w) at day 7 to 18,204 μg/g (1.8% w/w) at day 14 (Supplementary Fig. 2C). The level of total CBD increased 1.6-fold from 11,090 μg/g (1.1% w/w) at day 7 to 17,705 μg/g (1.7% w/w) at day 14 (Supplementary Fig. 2G). Additionally, the levels of CBD and THC increased 2.5-fold (699 μg/g; 0.06% w/w to 1740 μg/g; 0.1% w/w) and 4-fold (1,089 μg/g; 0.1% w/w to 4320 μg/g; 0.4% w/w), respectively (Supplementary Fig. 2D, F). However, other cannabinoids such as CBGA, CBG, THCA, and total THC remained unchanged during the first 2 weeks of flowering (Supplementary Fig. 2A, B, E, and H).

Leaf cannabinoids were also measured at the same time regime. The production of CBDA and THCA were 4868 μg/g (0.48% w/w) and 5220 μg/g (0.52% w/w), respectively at day 7. However, the concentrations did not change during the early flowering stage (Supplementary Fig. 2A–F).

These results suggest selective activation of flower cannabinoids biosynthetic genes. By deactivating the cannabinoid production in other tissues, presumably the catalytic energy and carbons used in leaf tissues were redirected to the flower organ for concentrating cannabinoid production in the specialized trichome cells. Supplementary Table 1 presents the measured floral cannabinoid concentrations of the immature flower tissues from control plants (day 1) and stress-treated plant (day 6 or day 8) that were used to calculate the production values during that timeframe.

These results are consistent with the previous studies that most cannabinoids production is organ and location–specific, with the trichome-dense flowers being the organs that produce the greatest amount of cannabinoids (Bernstein et al. 2019b). Another study also showed that cannabinoid production can vary greatly among different inflorescence locations with greater concentrations toward the apical meristem (Namdar et al. 2018).

### Cannabinoid productions in response to mechanical wounds

Field-grown *Cannabis* is constantly exposed to adverse biotic (e.g., microbial and herbivore pests), abiotic (e.g., excess heat, drought, and wind), and man-made stresses (e.g., tractor). These stresses have been reported to significantly impact cannabinoid production.

To investigate how mechanical damage affects cannabinoid metabolism, hemp clones (7 weeks old) were tested in grow tents located in a greenhouse. Treatments were started during the first week of flower to elucidate the greatest recordable amount of response, and to avoid the senescence phase of growth. Plants were harvested for the temporal- and spatial-specific cannabinoid analyses 14 days after the transition to the short-day, which limited this study to understanding the effects of these stresses on the onset of flowering; however, future studies should investigate the impact these stresses have on mature flower.

**Fig. 1:**
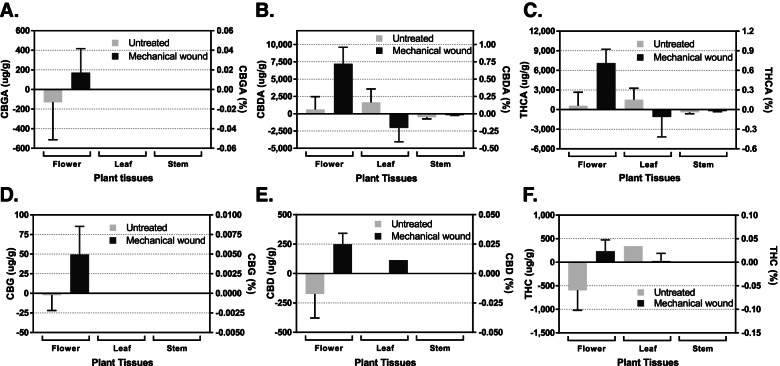
No impact on the production of cannabinoids in response to 5 days of short-term mechanical wounding during the first week of flowering. Quantitative comparisons of cannabigerolic acid (**A**), cannabidiolic acid (**B**), Δ-tetrahydrocannabinolic acid (**C**), cannabigerol (**D**), cannabidiol (**E**), and Δ-tetrahydrocannabinol (**F**) production in hemp flower, leaf, and stem tissues in response to 5 days of mechanical wound treatment. The values provided represent the difference between the cannabinoid concentration on day 1 and day 6 of flower. Negative values indicate that the day 6 concentration was lower than the concentration on day 1, whereas a positive value indicates an increase of that cannabinoid over the 6-day period. Statistical analysis was performed using one-way ANOVA, followed by Tukey’s multiple comparisons test. The bar graph represents mean ± s.d. (*n* = 3)

Figure 1 shows the changes of cannabinoid accumulation in three *Cannabis* tissues—immature flower, leaf, and stem—in response to mechanical wounding. The seven cannabinoid compounds described above were quantified using HPLC. The cannabinoid level observed after 5 days of treatment was compared to the 5-day chemical profile difference shown in the control plants. In the control plants, the cannabinoid production remained about the same level for 5 days. The mechanical wounding also did not impact the level of any cannabinoids (*n* = 3, *p* > 0.05) in any tissues of the plants (Fig. 1 and Table 1). In leaf and stem tissues, only CBDA and THCA were quantifiable and appeared not to be changed after mechanical wounding (*n* = 3, *p* > 0.05). Other cannabinoid compounds such as CBGA and CBG were under detectable limit in both treated and control plants.

**Table 1:** Summary of cannabinoid production per treatment

	Mechanical wound (5-day)		Herbivore (5-day)		Excess heat (7-day)		Drought (7-day)	
	Control (μg/g)	Treatment (μg/g)	Control (μg/g)	Treatment (μg/g)	Control (μg/g)	Treatment (μg/g)	Control (μg/g)	Treatment (μg/g)
**CBGA**	− 130 ± 663	172 ± 424	**308 ± 168**	− 24 ± 47**	**206 ± 147**	− 182 ± 133***	265 ± 103	34 ± 188
**CBG**	− 2 ± 28	49 ± 51	**69 ± 85**	− 52 ± 43*	**21 ± 29**	− 112 ± 105*	**336 ± 49**	622 ± 110***
**CBDA**	611 ± 3216	7223 ± 4113	671 ± 2108	− 1367 ± 1053	− 1948 ± 3065	− 3081 ± 1442	4131 ± 3065	826 ± 4417
**CBD**	− 174 ± 354	249 ± 162	**755 ± 397**	194 ± 121*	− 55 ± 174	− 338±471	**1182 ± 60**	297 ± 267****
**THCA**	593 ± 3596	7112 ± 3671	200 ± 2825	− 806 ± 911	− 1917 ± 2129	− 2829±718	3201 ± 4801	3849 ± 3851
**THC**	− 596 ± 734	232 ± 421	1771 ± 1034	687 ± 301	− 21 ± 398	− 1137 ± 1391	**3927 ± 242**	580 ± 555****

Floral cannabinoid production differences in the 2 weeks old immature buds in response to mechanical wound, herbivore, excess heat, and drought. Plants were harvested for the cannabinoid analysis 14 days after the transition to the short-day. For statistical analyses, three floral tissues from control- and stress-treated plants were quantified for cannabinoid production. The differences in the cannabinoid level were analyzed by one-way ANOVA, followed by Tukey’s multiple comparisons test. Significantly changed cannabinoid in the stress-treated immature buds are depicted as bold with asterisk in the table (*p < 0.05, **p < 0.01, ***p < 0.001, ****p < 0.0001). Note: concentration values reported are the difference between the concentration on day 1 and the concentration on day 6 or 8. A positive value indicates that an increase in that cannabinoid was observed in that 6–8 day period, whereas, a negative value indicates that cannabinoid decreased concentration

Several studies have revealed that short-term mechanical damage affects the production of secondary metabolites in cotton (Park et al. 2019b), *Catharanthus roseus* (Chen et al. 2018), sugar beet (Lafta and Fugate 2011), and lettuce (Saltveit 2000). These studies showed that the production of secondary metabolites (i.e., terpenoids, alkaloids, and phenolic compounds) that play an important role in direct defense were increased by the abiotic stress. Unlike these crops, short-term mechanical stress (1–5 days) did not affect the cannabinoid production in the studied hemp variety. While we did not study long-term effects of this mechanical damage, it appears that 5 days was not sufficient to induce any biosynthetic changes in the phytocannabinoid production. Future studies will be needed to understand the long-term effects on mature flower chemometrics; however, it does not appear to affect a hemps plant ability to produce cannabinoids at the onset of flower.

### Cannabinoid productions in response to herbivore stress

Insect pests can be classified by how they infest *Cannabis*, such as insects with “chewing mouth parts” affect the roots, leaves, stems, and flowers, and “piercing-sucking” insects can bypass insecticidal cannabinoids on the surface of the plant to access the sap within the plant (McPartland 1996). The vast nature of the types of insects that affect *Cannabis* cultivation provides numerous outcomes when infested and activates various stress responses depending on the insect. Thus, this investigation of the cannabinoid content response to particular insects is essential to field-grown hemp since cannabinoids are one of the most valued products of the *Cannabis* plant.

**Fig. 2:**
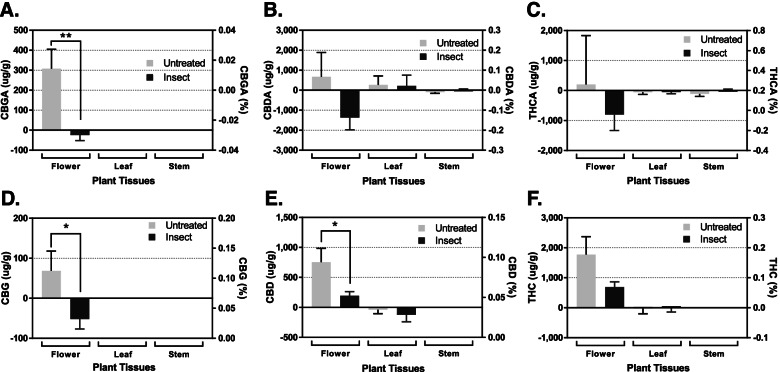
Differential cannabinoid production in response to 5 days of short-term herbivore stress during the first week of flowering. Quantitative comparisons of cannabigerolic acid (**A**), cannabidiolic acid (**B**), Δ-tetrahydrocannabinolic acid (**C**), cannabigerol (**D**), cannabidiol (**E**), and Δ-tetrahydrocannabinol (**F**) production in hemp flower, leaf, and stem tissues in response to 5 days of caterpillar larvae *M. sexta* foraging. The values above indicate the difference in concentrations between day 1 and day 6 of flowering. A negative value indicates a lower concentration after 6 days of flowering than at initiation, whereas a positive value indicates an increase in concentration over that 6-day period. Statistical analysis was performed using one-way ANOVA, followed by Tukey’s multiple comparisons test. The bar graph represents mean ± s.d. (*n* = 3). **p < 0.01 and *p < 0.05

To examine how the insect herbivores affect the cannabinoid production and composition, 20 3rd instar caterpillar larvae of tobacco hornworm *Manduca sexta* were placed on the 7-week-old hemp plants’ leaves for 5 days. It was observed that the larval insects preferentially feed on leaf tissues over stems, flowers, bracts, and petals. Figure 2 shows quantitative comparisons of 5 days of cannabinoid production between control and insect damaged hemp plants. The herbivore wounding significantly reduced the cannabinoid production of CBGA, with the control plants accumulating 308 μg/g (0.03% w/w) to the treated plants losing 24 μg/g (0.0024% w/w) (*p* < 0.01), CBG, accumulating 69 μg/g (0.0069% w/w) in control plants to losing 52 μg/g (0.0052% w/w) in treated plants (*p* < 0.05), and CBD, accumulating 755 μg/g (0.075% w/w) in control plants to accumulating 194 μg/g (0.019% w/w) in treated plants (*p* < 0.05) while other cannabinoids, CBDA, THCA, and THC levels (Fig. 2 and Table 1) remained unchanged during the 7-day observation window. While the reduction of CBGA, CBG and CBD are significant, the treatment procedure could be improved by replacing the porous screen with a non-light blocking insect containment; however, due to the minimal light interference (4.3% photosynthetically active radiation reduction) observed here, it suggests the changes that occurred were due solely to the insect pressure, although future studies should verify these results with differing insect contaminant approaches.

*Manduca sexta* caterpillar regurgitates onto the wounded site of a host plant while feeding (Paudel Timilsena and Mikó 2017). Insect regurgitant contains small molecular elicitors such as fatty acid conjugates, inceptin, and calliferins, as well as larger enzymatic molecules such as a glucose oxidase protein (Paudel Timilsena and Mikó 2017). The regurgitant is used to suppress plant defense mechanisms, leading to the changes in metabolic profiles that involve the release of toxic chemicals such as alkaloids, anthocyanins, phenols, and quinones, as well as volatile terpenoids that repel natural enemy insects (War et al. 2012). However, it was unexpected that the insect damage decreased cannabinoid production as they have been previously considered an effective stimulator to stress response (Benelli et al. 2018; Park et al. 2019a). The observed decrease is likely due to cannabinoid biosynthesis not being triggered by this particular herbivore’s stressors, the regurgitant suppressing the plant’s defense response, or the signal to increase production may not have been observable within the limited 5-day window of this study.

### Cannabinoid production in response to excess heat

**Fig. 3:**
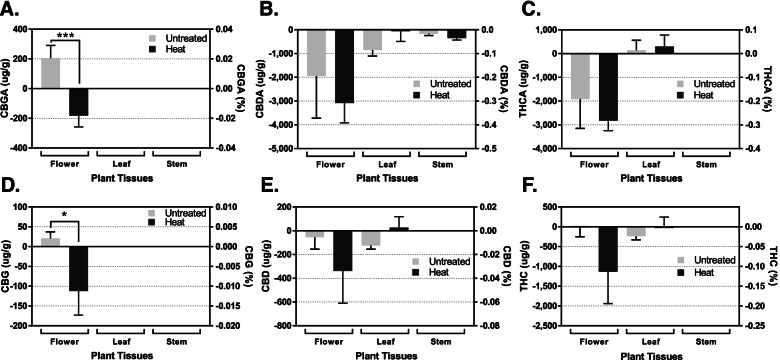
Differential cannabinoids production in response to 7 days of short-term heat stress during the first 2 weeks of flowering. Quantitative comparisons of cannabigerolic acid (**A**), cannabidiolic acid (**B**), Δ-tetrahydrocannabinolic acid (**C**), cannabigerol (**D**), cannabidiol (**E**), and Δ-tetrahydrocannabinol (F) production in hemp flower, leaf, and stem tissues in response to 7 days of heat (45–50 ^o^C) stress. The values above indicate the difference in concentrations between day 1 and day 8 of flowering. A negative value indicates a lower concentration after 8 days of flowering than at initiation, whereas a positive value indicates an increase in concentration over that 8-day period. Statistical analysis was performed using one-way ANOVA, followed by Tukey’s multiple comparisons test. The bar graph represents mean ± s.d. (*n* = 3). ***p < 0.001 and *p < 0.05

Hemp grows ideally at 24–30 °C in nitrogen-enriched fertilized soils (pH 6.0–7.5) under a regime of 16–24 h of light and 0–8 h of darkness with 40–60% humidity level (Adesina et al. 2020; Chandra et al. 2017). In fields, higher temperatures (> 31 °C) are a common stressor that alters plant physiology and metabolism. To examine how excess heat affects cannabinoid production, six hemp clones were heat-treated at 45–50 °C over 7 days and six clones served as controls at 22–27 °C. Figure 3 shows quantitative comparisons of cannabinoid production between control and heat-treated hemp plants over the 7-day period. Within 3 days, the hemp plants were completely wilted under excess heat regardless of the water supply ( 1L/day). It should be noted that plants were also exposed to water stress in addition to the heat stress due to the increased transpiration caused by the increased temperature. The HPLC demonstrated that excess heat caused significant cannabinoid metabolic changes. In the untreated control plants, female inflorescence produced 206 μg/g (0.02% w/w) of CBGA and 21 μg/g (0.0021% w/w) of CBG during the 7-day observation window. After 7 days of excessive heat treatment, the concentration of CBGA and CBG decreased by 182 μg/g (0.0182% w/w) (*n* = 3, *p* < 0.001 and *p* < 0.05) and by 112 μg/g (0.0112% w/w) (*n* = 3, *p* < 0.05), respectively (Fig. 3 and Table 1). Contrastingly, CBDA, THCA, CBD, and THC bioaccumulations were not changed during this 7-day window (Fig. 3 and Table 1).

As with all cannabinoids, it was expected to see an increase in concentration over the 7-day period; however, heat stress caused the opposite effect. With the decreased concentrations of CBGA and CBG, it would be hypothesized that the enzymatic conversion of CBGA to THCA and CBDA would be happening at a faster rate in the treated plants causing an increase of the terminal cannabinoid concentrations while depleting the precursor pool. However, no significant difference was observed in the concentrations of THCA and CBDA, thus it appears the enzymatic conversion was not affected and the biosynthesis of CBGA may have been downregulated or inhibited upstream in the cannabinoid biosynthetic pathway. While we cannot definitively say whether it was the heat stress or watering habits that affected this cannabinoid production, this result does provide support for a potential downregulation of CBGA production during the initiation of flowering, which would be hypothesized to decrease overall cannabinoid production if the study would have observed longer into the flowering stage.

### Cannabinoid productions in response to drought

**Fig. 4:**
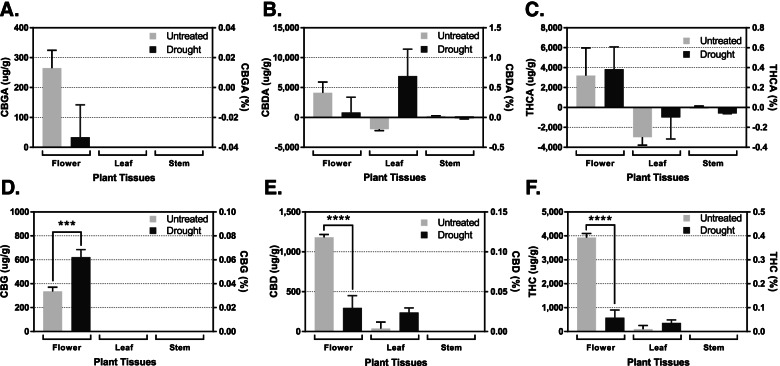
Differential cannabinoids production in response to 7 days of short-term drought stress during the first 2 weeks of flowering. Quantitative comparisons of cannabigerolic acid (**A**), cannabidiolic acid (**B**), Δ-tetrahydrocannabinolic acid (**C**), cannabigerol (**D**), cannabidiol (**E**), and Δ-tetrahydrocannabinol (**F**) production in hemp flower, leaf, and stem tissues in response to 7 days of drought stress. The values above indicate the difference in concentrations between day 1 and day 8 of flowering. A negative value indicates a lower concentration after 8 days of flowering than at initiation, whereas a positive value indicates an increase in concentration over that 8-day period. Statistical analysis was performed using one-way ANOVA, followed by Tukey’s multiple comparisons test. The bar graph represents mean ± s.d. (*n* = 3). ****p < 0.0001 and ***p < 0.001

Figure 4 and Table 1 show the cannabinoid production in response to 7 days of drought stress in the various hemp tissues. Notably, the drought treatment has changed the biosynthesis of CBG, CBD, and THC. Unlike other stresses investigated, drought significantly increased CBG production by 40% while CBD and THC were dramatically reduced by 70–80%. The control plants accumulated 336 μg/g (0.03% w/w) of CBG during the 8-day window, whereas, the amount accumulated in the treated plants significantly increased to 622 μg/g (0.06% w/w) (*n* = 3, *p* < 0.001). In addition, two other downstream products, CBD and THC concentrations were significantly decreased from accumulating 1182 μg/g (0.12% w/w) of CBD in the control plants to only accumulating 296 μg/g (0.02% w/w) of CBD in the plants subjected to drought stress (*n* = 3, *p* < 0.0001). Similarly, the control plants produced 3927 μg/g (0.39% w/w) of THC during this 8-day period, whereas the plants subjected to drought only accumulated 580 μg/g (0.05% w/w) of THC (*n* = 3, *p* < 0.0001).

CBG is a shared precursor molecule to the production of both CBD and THC. The increase of CBG accumulation might indicate the blockage of conversion into the two downstream intermediates, CBDA and THCA, resulting in accumulation of CBGA that was decarboxylated to CBG under these stress conditions. The catalytic enzymes, CBDA and THCA synthases may have malfunctioned enzymatically and/or their genes’ expression was downregulated resulting in less enzyme available. The reduced enzymatic activity consequently resulted in the decrease of downstream end-products (CBD and THC) while the precursor CBG levels accumulate. Water deficit is directly related to a variety of cellular processes including carbohydrate transport and metabolism, signal transduction mechanisms, and secondary metabolite biosynthesis, transport, and catabolism (Gao et al. 2018). Similar to their findings, in this study, drought stress resulted in the decreased levels of end-product cannabinoids.

Additional work should investigate if drought also affects other secondary metabolites such as terpenoids by measuring terpenoid content in response to drought stress, as well as time-specific drought stresses throughout the flower stage to determine the overall effects on cannabinoid concentration.

## Conclusion

Cannabinoid analyses supported previous studies that floral cannabinoid production is at least 2.5-fold greater than in the leaf tissues. Cannabinoid production at the onset of flowering is negatively associated with herbivory, excess heat, and drought while mechanical wounding showed no impact. These common field stresses are adequate to trigger changes in the cannabinoid profiles at the early flowering stage, especially drought which is the most impactful restraint for hemp growth in terms of cannabinoid production (70–80% reduction in CBD and THC). Further studies are necessary to validate if the observed changes apply to mature buds and understand the hypothesized underlying gene regulation causing these increases or decreases in cannabinoid concentrations. This study provides an initial understanding of the effects of biotic and abiotic stresses common when initiating flowering in hemp, as well as providing an understanding of the stresses that are most impactful to the onset of flowering to guide future studies to determine these various stresses’ effects, if they exists, on the chemical profile of mature hemp flowers.

## Supplementary Information

**Additional file 1: Supplementary Figure 1.** Tissue-specific cannabinoid production comparison. Tissue-specific cannabinoid production in the immature buds sampled on day 6 after the transition to the short-day that initiated flowering. Three plant tissues including flower, leaf, and stems were used in high pressure liquid chromatography to quantify cannabinoids including cannabigerolic acid, cannabidiolic acid, Δ-tetrahydrocannabinolic acid, cannabigerol, cannabidiol, and Δ-tetrahydrocannabinol. For statistical analyses, a total of 19-24 samples collected from flower, leaf, and stem tissues were compared by one-way ANOVA, followed by Tukey’s multiple comparisons test (***p < 0.001, ****p < 0.0001). **Supplementary Figure 2.** Time-specific cannabinoid production. Time course of cannabinoid production in immature buds and leaves at the first two weeks of flowering. Quantitative comparisons of cannabigerolic acid (A), cannabidiolic acid (B), Δ^9^-tetrahydrocannabinolic acid (C), cannabigerol (D), cannabidiol (E), Δ^9^-tetrahydrocannabinol (F), total cannabidiol (G), and total Δ^9^-tetrahydrocannabinol (H) production in immature buds and leaves were conducted using high pressure liquid chromatography. For statistical analyses, a total of 3-24 flower and leaf tissues collected on day 7, 12, and 14 after the transition to the short-day were compared by two-way ANOVA, followed by Tukey’s multiple comparisons test (*p < 0.05, **p < 0.01, ***p < 0.001, ****p < 0.0001). **Supplementary Table 1.** Time-specific floral cannabinoid concentrations per treatment. Floral cannabinoid concentrations in immature buds in the control groups and in response to mechanical wounding, herbivory, excess heat, or drought stresses. Concentration values in the table are the average of 2-3 hemp plants with standard deviation from the sampling on day 1 and day 6 or day 8 depending on whether the treatment was 5-days or 7-days respectively.

## Data Availability

All data generated or analyzed in this study are included in this published article.

## Abbreviations

CBGA: Cannabigerolic acid
CBG: Cannabigerol
CBDA: Cannabidiolic acid
CBD: Cannabidiol
THCA: Δ-Tetrahydrocannabinolic acid
THC: Δ-Tetrahydrocannabinol
CBN: Cannabinol
