# Supplementary material for: Effects of short-term environmental stresses on the onset of cannabinoid production in young immature flowers of industrial hemp (Cannabis sativa L.)
Source: J Cannabis Res. 2022 Jan 4;4:1. doi: 10.1186/s42238-021-00111-y (PMC8725245; doi:10.1186/s42238-021-00111-y)
Supplement: Supplementary file 1 — Additional file 1: Supplementary Figure 1. Tissue-specific cannabinoid production comparison. Tissue-specific cannabinoid production in the immature buds sampled on day 6 after the transition to the short-day that initiated flowering. Three plant tissues including flower, leaf, and stems were used in high pressure liquid chromatography to quantify cannabinoids including cannabigerolic acid, cannabidiolic acid, Δ-tetrahydrocannabinolic acid, cannabigerol, cannabidiol, and Δ-tetrahydrocannabinol. For statistical analyses, a total of 19-24 samples collected from flower, leaf, and stem tissues were compared by one-way ANOVA, followed by Tukey’s multiple comparisons test (***p < 0.001, ****p < 0.0001). Supplementary Figure 2. Time-specific cannabinoid production. Time course of cannabinoid production in immature buds and leaves at the first two weeks of flowering. Quantitative comparisons of cannabigerolic acid (A), cannabidiolic acid (B), Δ9-tetrahydrocannabinolic acid (C), cannabigerol (D), cannabidiol (E), Δ9-tetrahydrocannabinol (F), total cannabidiol (G), and total Δ9-tetrahydrocannabinol (H) production in immature buds and leaves were conducted using high pressure liquid chromatography. For statistical analyses, a total of 3-24 flower and leaf tissues collected on day 7, 12, and 14 after the transition to the short-day were compared by two-way ANOVA, followed by Tukey’s multiple comparisons test (*p < 0.05, **p < 0.01, ***p < 0.001, ****p < 0.0001). Supplementary Table 1. Time-specific floral cannabinoid concentrations per treatment. Floral cannabinoid concentrations in immature buds in the control groups and in response to mechanical wounding, herbivory, excess heat, or drought stresses. Concentration values in the table are the average of 2-3 hemp plants with standard deviation from the sampling on day 1 and day 6 or day 8 depending on whether the treatment was 5-days or 7-days respectively. [file 42238_2021_111_MOESM1_ESM.docx]

**Supplementary figure 1.** Tissue-specific cannabinoid production comparison. Tissue-specific cannabinoid production in the immature buds sampled on day 6 after the transition to the short-day that initiated flowering. Three plant tissues including flower, leaf, and stems were used in high pressure liquid chromatography to quantify cannabinoids including cannabigerolic acid, cannabidiolic acid, Δ⁹-tetrahydrocannabinolic acid, cannabigerol, cannabidiol, and Δ⁹-tetrahydrocannabinol. For statistical analyses, a total of 19-24 samples collected from flower, leaf, and stem tissues were compared by one-way ANOVA, followed by Tukey’s multiple comparisons test (****p*<0.001, *****p*<0.0001).


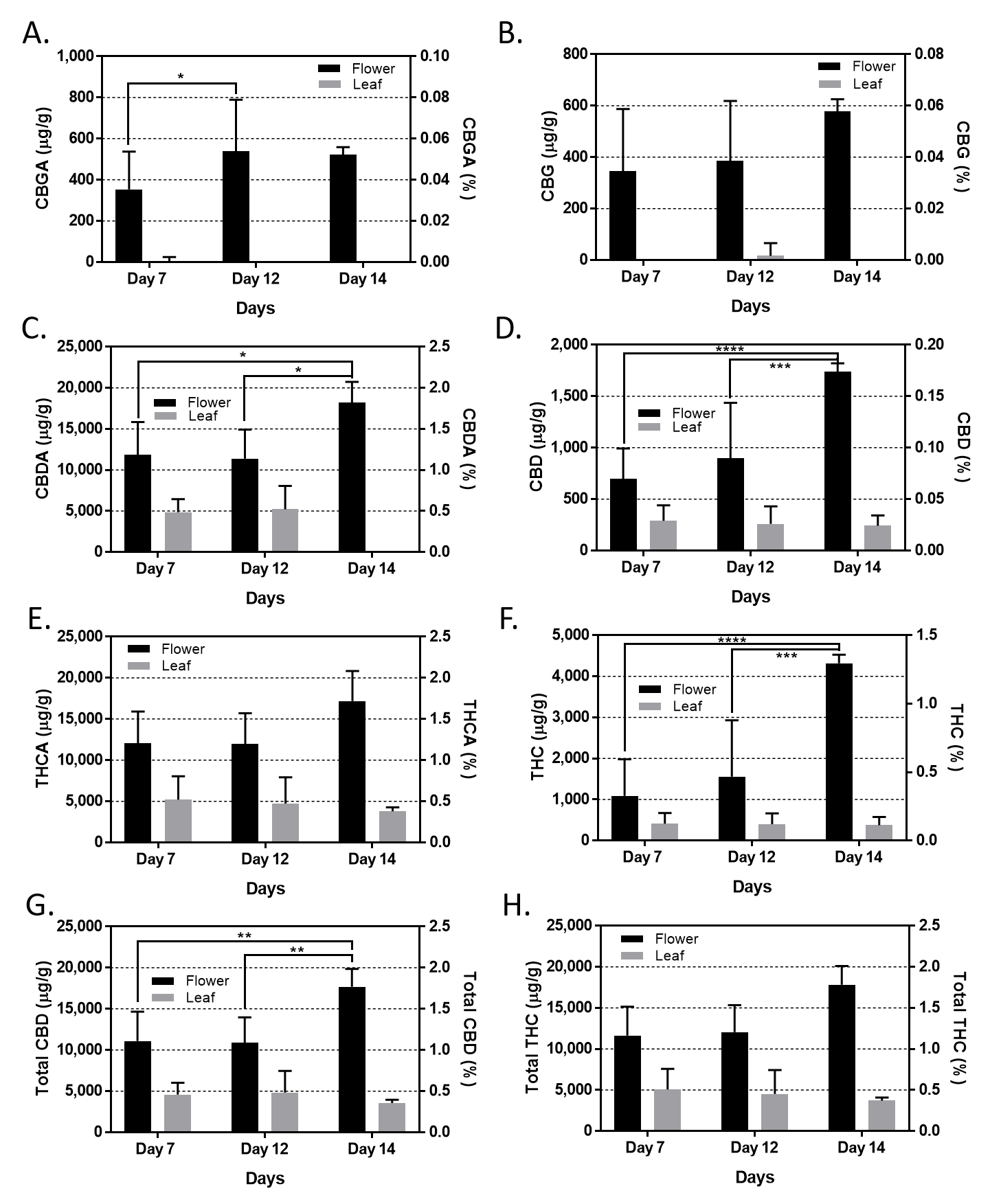


**Supplementary figure 2.** Time-specific cannabinoid production. Time course of cannabinoid production in immature buds and leaves at the first two weeks of flowering. Quantitative comparisons of cannabigerolic acid (A), cannabidiolic acid (B), Δ⁹-tetrahydrocannabinolic acid (C), cannabigerol (D), cannabidiol (E), Δ⁹-tetrahydrocannabinol (F), total cannabidiol (G), and total Δ⁹-tetrahydrocannabinol (H) production in immature buds and leaves were conducted using high pressure liquid chromatography. For statistical analyses, a total of 3-24 flower and leaf tissues collected on day 7, 12, and 14 after the transition to the short-day were compared by two-way ANOVA, followed by Tukey’s multiple comparisons test (**p*<0.05, ***p*<0.01, ****p*<0.001, *****p*<0.0001).

**Supplementary table 1.** Time-specific floral cannabinoid concentrations per treatment. Floral cannabinoid concentrations in immature buds in the control groups and in response to mechanical wounding, herbivory, excess heat, or drought stresses. Concentration values in the table are the average of 2-3 hemp plants with standard deviation from the sampling on day 1 and day 6 or day 8 depending on whether the treatment was 5-days or 7-days respectively.

|  | **Mechanical wound (5-day)** | | | | **Herbivore (5-day)** | | | | **Excess heat (7-day)** | | | | **Drought (7-day)** | | | |
| --- | --- | --- | --- | --- | --- | --- | --- | --- | --- | --- | --- | --- | --- | --- | --- | --- |
|  | Control | | Treated | | Control | | Treated | | Control | | Treated | | Control | | Treated | |
|  | Day 1  (μg/g) | Day 6  (μg/g) | Day 1  (μg/g) | Day 6  (μg/g) | Day 1  (μg/g) | Day 6  (μg/g) | Day 1  (μg/g) | Day 6  (μg/g) | Day 1  (μg/g) | Day 8  (μg/g) | Day 1  (μg/g) | Day 8  (μg/g) | Day 1  (μg/g) | Day 8  (μg/g) | Day 1  (μg/g) | Day 8  (μg/g) |
| **CBGA** | 566±241 | 436±429 | 271±39 | 443±421 | 215±90 | 523±124 | 152±58 | 128±16 | 448±20 | 654±134 | 541±157 | 359±93 | 256±80 | 521±37 | 382±187 | 416±72 |
| **CBG** | 159±24 | 168±48 | 132±19 | 199±38 | 349±79 | 418±57 | 314±23 | 262±22 | 607±41 | 628±32 | 542±36 | 430±72 | 238±17 | 579±47 | 187±28 | 809±105 |
| **CBDA** | 10,271±2819 | 10,883±5492 | 8,251±3,194 | 15,474±1,213 | 8,683±2,914 | 9,354±980 | 8,352±989 | 6,984±178 | 15,865±1,586 | 13,917±1,612 | 12,449±526 | 9,368±1349 | 14,073±1,390 | 18,204±2,533 | 16,842±5,003 | 17,668±2,597 |
| **CBD** | 577±119 | 402±240 | 406±93 | 655±97 | 739±25 | 1494±412 | 531±56 | 724±96 | 844±29 | 790±182 | 1187±528 | 849±401 | 558±50 | 1740±80 | 753±234 | 1049±129 |
| **THCA** | 10,402±2,157 | 10,995±5,527 | 8,052±2738 | 15,165±1,012 | 9,413±2,885 | 9,613±84 | 8,756±1,022 | 7,950±317 | 17,158±1688 | 15,241±727 | 12,429±681 | 9,599±283 | 13,992±1,514 | 17,193±3,656 | 16,025±5,003 | 19,874±1,784 |
| **THC** | 945±619 | 349±173 | 419±392 | 651±88 | 1,313±287 | 3,085±1,225 | 676±92 | 1,362±377 | 1,274±88 | 1,253±348 | 2,699±1,548 | 1,562±831 | 367±51 | 4,320±208 | 1,019±598 | 1,599±173 |
